# An integrated genetic map based on four mapping populations and quantitative trait loci associated with economically important traits in watermelon (*Citrullus lanatus*)

**DOI:** 10.1186/1471-2229-14-33

**Published:** 2014-01-20

**Authors:** Yi Ren, Cecilia McGregor, Yan Zhang, Guoyi Gong, Haiying Zhang, Shaogui Guo, Honghe Sun, Wantao Cai, Jie Zhang, Yong Xu

**Affiliations:** 1 Institute of Vegetables and Flowers, Chinese Academy of Agricultural Sciences, Beijing, China; 2 National Engineering Research Center for Vegetables, Beijing Academy of Agriculture and Forestry Sciences, Key Laboratory of Biology and Genetic Improvement of Horticultural Crops (North China), Beijing 100097, China; 3 Department of Horticulture and Institute of Plant Breeding, Genetics and Genomics, University of Georgia, Athens, GA 30602, USA

**Keywords:** Watermelon, Integrated genetic map, QTL, Sugar content

## Abstract

**Background:**

Modern watermelon (*Citrullus lanatus L.*) cultivars share a narrow genetic base due to many years of selection for desirable horticultural qualities. Wild subspecies within *C. lanatus* are important potential sources of novel alleles for watermelon breeding, but successful trait introgression into elite cultivars has had limited success. The application of marker assisted selection (MAS) in watermelon is yet to be realized, mainly due to the past lack of high quality genetic maps. Recently, a number of useful maps have become available, however these maps have few common markers, and were constructed using different marker sets, thus, making integration and comparative analysis among maps difficult. The objective of this research was to use single-nucleotide polymorphism (SNP) anchor markers to construct an integrated genetic map for *C. lanatus.*

**Results:**

Under the framework of the high density genetic map, an integrated genetic map was constructed by merging data from four independent mapping experiments using a genetically diverse array of parental lines, which included three subspecies of watermelon. The 698 simple sequence repeat (SSR), 219 insertion-deletion (InDel), 36 structure variation (SV) and 386 SNP markers from the four maps were used to construct an integrated map. This integrated map contained 1339 markers, spanning 798 cM with an average marker interval of 0.6 cM. Fifty-eight previously reported quantitative trait loci (QTL) for 12 traits in these populations were also integrated into the map. In addition, new QTL identified for brix, fructose, glucose and sucrose were added. Some QTL associated with economically important traits detected in different genetic backgrounds mapped to similar genomic regions of the integrated map, suggesting that such QTL are responsible for the phenotypic variability observed in a broad array of watermelon germplasm.

**Conclusions:**

The integrated map described herein enhances the utility of genomic tools over previous watermelon genetic maps. A large proportion of the markers in the integrated map are SSRs, InDels and SNPs, which are easily transferable across laboratories. Moreover, the populations used to construct the integrated map include all three watermelon subspecies, making this integrated map useful for the selection of breeding traits, identification of QTL, MAS, analysis of germplasm and commercial hybrid seed detection.

## Background

Watermelon (*Citrullus lanatus* L.) is an important specialty crop accounting for approximately 7% of the world agricultural area devoted to vegetable crops. China is the largest producer and consumer, with an annual production of about 68 million tons (http://faostat.fao.org). The long term cultivation and selection of watermelon for desirable horticultural qualities resulted in modern watermelon cultivars with a narrow genetic base and susceptibility to a large number of diseases and pests [1]. Watermelon includes three subspecies: *C. lanatus* subsp*. lanatus* L., which represents a group of ancient cultigens, the ‘tsamma’ or ‘citron’ watermelon, that naturally thrives in southern Africa; *C. lanatus* subsp. *mucosospermus* L., which represents the egusi watermelon group that has large edible seeds with a fleshy pericarp [2]; and *C. lanatus* subsp. *vulgaris* L., which represents the sweet (dessert) watermelon group that gave rise to the modern cultivated elite watermelon [3]. The citron and egusi types are sources of resistance to economically important diseases, such as Fusarium wilt races 0, 1, and 2 (PI 296341-FR; citron) [4] and *zucchini yellow mosaic virus* (ZYMV; PI 595203; egusi) [5]. However, several critical steps that include accurate phenotyping for disease or pest resistance, high density genetic mapping and genome sequencing and assembly studies are needed as part of continuous efforts to utilize citron and egusi type germplasm for the improvement of elite watermelon cultivars. The construction of highly saturated maps is often a time-consuming process, especially if investigators are employing different parental material and markers are not easily transferable. Merged maps are attractive since their integration allows for increased marker density. A number of integrated linkage maps have been developed in economically important crops to increase marker density and integrate the QTL information, including melon (*Cucumis melo* L.) [6], grapevine (*Vitis vinifera* L.) [7], lettuce (*Lactuca sativa* L.) [8], maize (*Zea mays* L.) [9], sorghum (*Sorghum bicolor* L.) [10], red clover (*Trifolium pratense* L.) [11], ryegrass (*Lolium ssp* L.) [12] and wheat (*Triticum aestivum* L.) [13].

Watermelon has a genome size of 425 Mb (2*n* = 2*x* = 22 [14]). Several genetic linkage maps have been constructed for the crop however these maps often have a much higher number of linkage groups than the expected 11, with uneven marker distribution. These low density maps were mainly based on isozymes [15,16], RAPD (randomly amplified polymorphic DNA), RFLP (restriction fragment length polymorphism), AFLP (amplified fragment length polymorphism) and SRAP (sequence related amplified polymorphism) markers, and only a limited number of SSR (simple sequence repeat) markers [17-19]. More recently, a high density linkage map with 953 loci was constructed using simple sequence repeat (SSR), insertion-deletion (InDel) and structure variation (SV) markers [20] by the National Engineering Research Center for Vegetables, and the first single-nucleotide polymorphism (SNP) genetic maps were constructed through a collaboration between the University of Georgia (Athens, GA) and Monsanto (Woodland, CA), resulting in 388 public SNP markers [21]. Quantitative trait loci (QTL) associated with economically important seed and fruit traits in watermelon were also mapped in the three individual populations used to create the SNP maps [21-23]. Together, these maps represent all three subspecies of *C. lanatus*, however the maps produced by the two research groups don’t include any common markers and possess large numbers of individual-specific markers. This complicates comparisons of colinearity, segregation distortion and QTL locations across the three *C. lanatus* subspecies. The SNP maps also have more than 11 linkage groups and some large gaps (>20 cM). The construction of an integrated map provides the opportunity to merge the SNP maps used to map horticulturally important traits and the 97103 × PI 296341-FR RIL map used to anchor the watermelon genome. The integration of the four maps representing the three subspecies will make it possible to compare and confirm marker order [10] and QTL information. The integrated map also provides more choice in the type of marker and increases the probability of polymorphic markers in important chromosomal intervals and greater genome coverage than single crosses.

Here, we report the colinearity among four genetically diverse watermelon maps and the construction of an integrated linkage map of watermelon. We also report new QTL identified for fruit sugar traits and the location of previously reported QTL [21-23] on the integrated map. This made it possible to compare the genomic location of the QTL mapped in the different populations and suggest a common nomenclature to name these QTL.

## Results and discussion

### Construction of the integrated map

**Figure 1:**
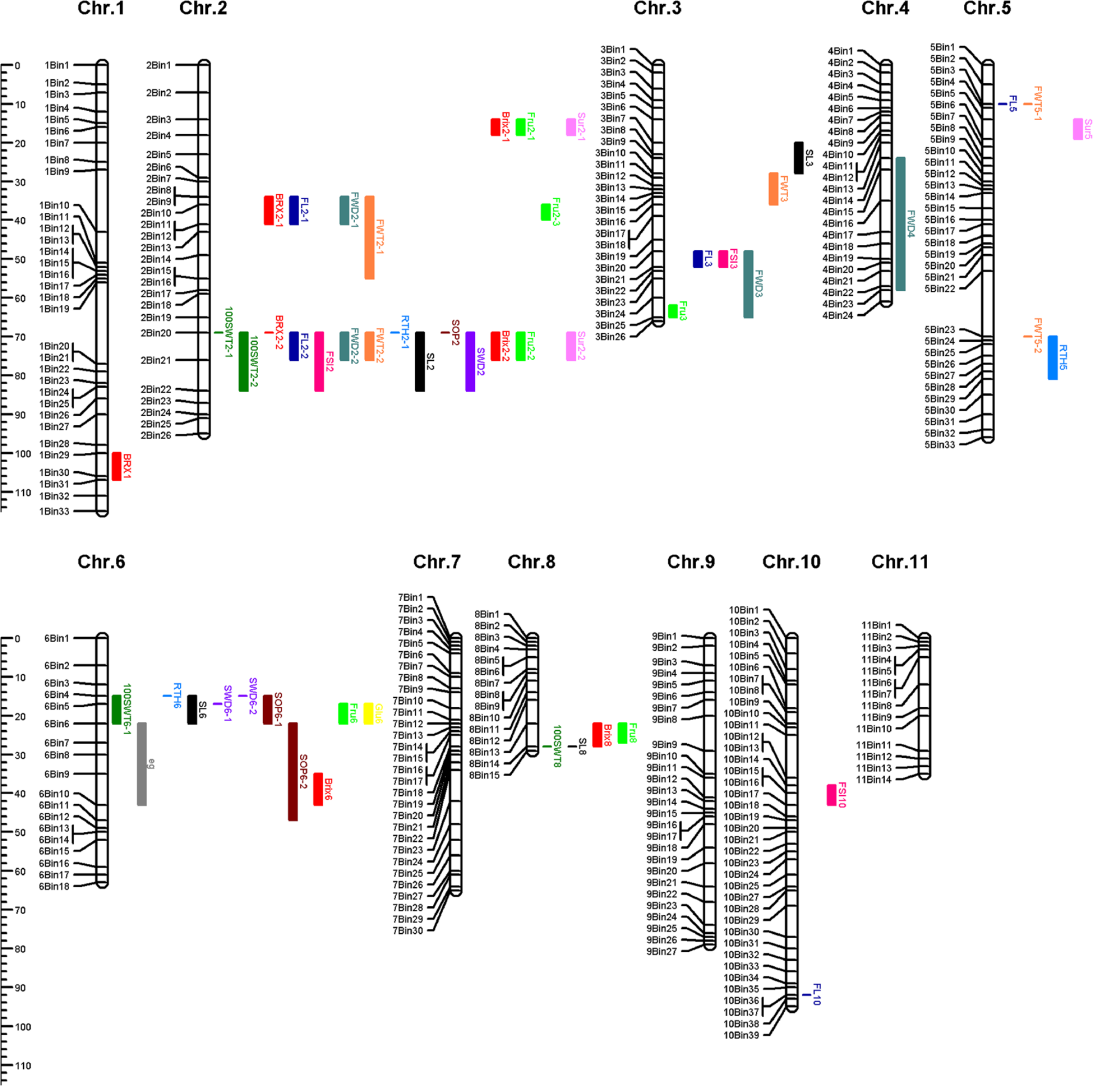
**Integrated watermelon genetic map and QTL position.** Chromosome 1 to 11 of watermelon are according to Ren et al. [20]. The map distance is given on the left in centimorgans (cM) from the top of each chromosome.QTL are located in a skeleton bins of the integrated map chromosomes 1 to 11. QTL are designated according to Additional file 1: Table S5, QTL’s positions were defined by the support interval. Skeleton bins of each chromosome are indicated as black dashes. Brix (BRX), fructose(Fru), sucrose (Suc), glucose (Glu), fruit weight (FWT), fruit length (FL), fruit width (FWD), fruit shape index (FSI), rind thickness (RTH), 100 seed weight (100SWT), seed length(SL), seed width (SWD), seed oil percentage (SOP) and egusi (eg) seed loci are represented by different colours. The brix loci were identified in this research while the BRX loci were identified by previous research [21].

In order to make map integration possible, the 388 SNP markers described by Sandlin et al. [21] were genotyped in the 97103 × PI 296341-FR RIL population [20] by mining genotyping by sequencing (GBS) data for the population. A total of 386 SNP loci were segregating in the latter population, allowing the integration of 244449 data points generated from four mapping populations representing eight watermelon parental accessions (Table 1). The largest data sets were from the parental accession 97103 × PI 296341-FR RIL population. Based on the high density saturated genetic map [20] and SNP map [21], we integrated 698 SSR, 219 InDel, 36 SV and 386 SNP markers to construct the integrated map. The integrated map contains 1339 markers, spanning 798 cM with an average marker interval of 0.6 cM (Figure 1, Table 2). The size of the watermelon genome is 425 Mbp [14] and the map defined herein represents average physical intervals of 317 Kb per marker, making it the most saturated map of watermelon to date. The previous consensus map by Sandlin et al. [21] for the three SNP maps contained 380 markers with a total length of 1753 cM and an average marker interval of 4.6 cM. The Sandlin et al. [21] map was also based on comparisons of marker order and distances, rather than recombination frequencies.

**Table 1:** Summary of component mapping population used to construct the watermelon integrated map

** Population code**	**Parental lines**	**Subspecies**	**Population type**	**Population size**	**Number of markers**	**Map length (cM)**	**Marker type**	**Reference**
97103 × PI 296341-FR	97103 PI 296341-FR	*vulgaris lanatus*	RIL	103	953	800	SSR InDel SV	[20]
ZWRM × citron	ZWRM50 PI 244019	*vulgaris lanatus*	F_2_	182	200	1,144	SNP	[21]
KBS × NHM	Klondike Black Seeded	*vulgaris*	RIL	164	222	1,438	SNP	[21]
	New Hampshire Midget	*vulgaris*						
SII × egusi	Strain II PI 560023	*vulgaris mucosospermus*	F_2_	187	210	1,514	SNP	[21]

**Table 2:** Summary of the distribution of genetic markers in watermelon integrated genetic map

**Chr.s**	**No. loci**	**No. SSRs**	**No. InDels**	**No. SNPs**	**No. SVs**	**Genetic distance (cM)**	**Marker density (cM/marker)**
**Chr.1**	159	77	32	45	5	115.4	0.7
**Chr.2**	154	92	14	47	1	94.8	0.6
**Chr.3**	124	66	23	34	1	65.2	0.5
**Chr.4**	90	50	14	19	7	60.5	0.7
**Chr.5**	143	68	26	45	4	96.4	0.7
**Chr.6**	106	58	17	27	4	63.3	0.6
**Chr.7**	112	44	27	38	3	63.9	0.6
**Chr.8**	90	53	10	27	0	29.2	0.3
**Chr.9**	133	65	23	42	3	79.1	0.6
**Chr.10**	120	61	24	29	6	94.9	0.8
**Chr.11**	108	64	9	33	2	35.3	0.3
**Total**	1339	698	219	386	36	798.0	0.6

SSR: simple sequence repeat, InDel: insertion-deletion, SNP: single-nucleotide polymorphism, SV: structure variation.

The largest number of markers (159) was placed on chromosome 1 which also had the longest genetic distance of 115.4 cM, while chromosome 8 only has 90 markers spanning 29.2 cM, making it the smallest and shortest chromosome. The marker information for the mapped SSR, InDel, SV, SNP markers and corresponding scaffolds, as well as the physical location of the markers on the draft genome sequence are listed in Additional file 1: Table S1.

### Colinearity among individual maps, integrated map and physical map

**Figure 2:**
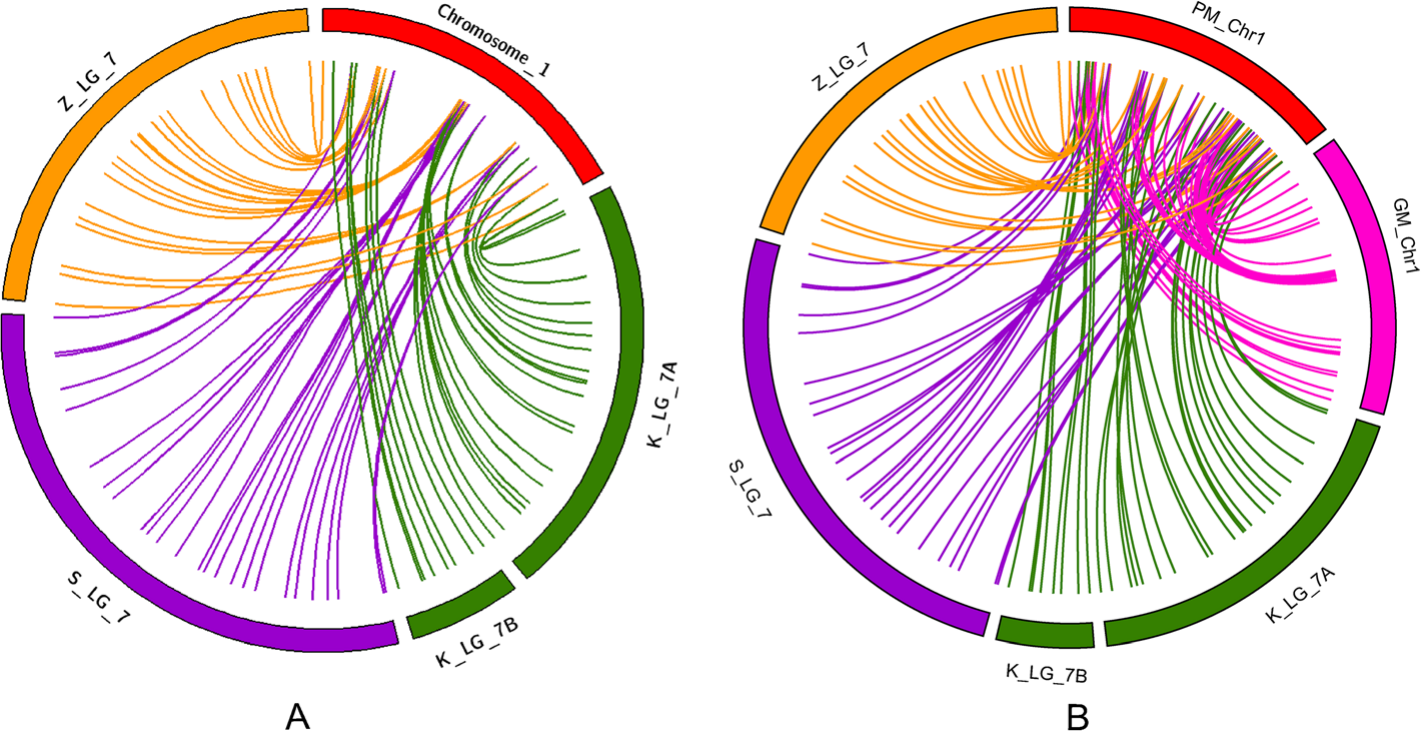
**Colinearity of locus in chromosome 1. (A)** Colinearity of marker’s order in individual and integrated watermelon genetic maps in chromosome 1. **(B)** Colinearity of locus order among three populations based genetic maps, integrated watermelon genetic map (GM) and physical map (PM) in chromosome 1. Loci that are common between pairs of populations are connected by lines. Population codes K_LG, S_LG, Z_LG corresponding to KBS × NHM, StrainII × PI 560023 (egusi), ZWRM50 × PI 244019 (citron) genetic maps [21]. Chromosome No. refer to the integrated 97103 × PI296341-FR map [20].

A total of 386 SNP markers were mapped in the 97103× PI 296341-FR population. Because there were only 45 common SNP markers among the four mapping populations, we chose individual SNP maps to do colinearity comparison with the integrated map. There were 216, 203 and 200 common markers with the integrated map respectively in KBS × NHM, SII × egusi, and ZWRM50 × citron map. There was a high degree of marker colinearity between the four individual maps for all the markers in common with the integrated map (Figure 2 and Additional file 2: Figure S1). The same colinearity was observed between the integrated genetic map (GM) and physical map (PM) (Figure 2 and Additional file 2: Figure S1). Only in the region 50.7 cM to 55.1 cM of chromosome 1 was there an obvious reverse of the marker order between the 97103× PI 296341-FR map and the three individual Sandlin et al. [21] maps. However, since this region on chromosome 1 has a high degree of marker colinearity in the three individual SNP maps, this reverse may due to the difference in calculating methods during the genetic mapping process. The lack of differences in locus order suggests that major chromosomal rearrangements have not occurred during the recent evolutionary history (i.e., domestication) of watermelon species.

### Marker segregation distortion analysis among individual maps

Segregation distortion is prevalent in wide-cross populations, and plays an important role in plant genome evolution [24]. Nine significant segregation distortion regions (SDRs; p < 0.001; highlighted regions in Additional file 1: Table S1) were detected in the elite cultivar × wild citron (97103 × PI 296341-FR) RIL population [20]. The marker alleles from the cultivar parent (97103) were favored in the SDR on chromosomes 1, 2, 3, 4, 5, 7, 8, 9 and 11 while the citron (PI 296341-FR) alleles were favored in the SDR on chromosome 10 . In the ZWRM50 (elite) × PI 244019 (citron) F_2_ population, five SDRs (highlighted regions in Additional file 1: Table S4) were detected (p < 0.001). Although both populations had SDRs on chromosomes 1, 2, 5 and 10, they were in different regions of the chromosomes. The markers on chromosome 10 are skewed towards the citron parent (PI 296341-FR and PI 244019) in both populations, while markers on chromosome 5 are skewed towards the cultivar parent (line 97103 and ZWRM50) in both populations. However, markers on chromosomes 1, 2 and 11 skewed toward different type parents in the two populations. No SDRs were detected in the crosses between two elite cultivars, Klondike Black Seeded × New Hampshire Midget (KBS × NHM), and an elite cultivar and wild egusi accession, Strain II × PI 560023 (SII × Egusi).

The use of inter-specific hybrids to construct genetic maps is a common strategy to ensure the availability of a high number of polymorphic markers, and in such cases segregation distortion may be common [25]. Depending on the relative frequency and intensity of the segregation distortion, it may not interfere in the map construction. Nevertheless, such distortion, especially in the regions skewed toward the wild genotypes on chromosome 10, may hinder the transfer of economically important alleles during watermelon breeding. The relatively high number of genomic regions with skewed segregation towards the elite parent detected in the 97103 × PI 296341-FR RIL population reinforces the hypothesis that such distortion likely originated from unintentional selection of genes in the elite parent that may be associated with fruit and seed production (especially ease of production) during RIL line development [20]. Given that SDRs were not detected in the crosses between two elite cultivars and the elite × egusi type, the introgression of novel, economically important alleles from exotic watermelon germplasm into elite modern cultivars should be relatively unimpeded by the use of elite × egusi type crosses during watermelon improvement.

### Phenotypic performance of the 97103 × PI 296341-FR population for sugar content traits

The elite line 97103 had higher values than PI 296341-FR for all four sugar content traits measured (Table 3). Transgressive segregation was observed in the RIL population for fructose, glucose and sucrose content, while the averages for soluble solid content (brix) and sucrose were skewed towards the citron parent. Shifts of mean brix values towards the citron parent have been observed previously in elite × citron populations [21]. The total sucrose content in the RIL population was much lower than the elite parent (97103), probably because the sucrose is broken down by invertase into fructose and glucose [26] in the RIL population leading to fructose-accumulating fruit types. The heritability (*h*^*2*^) of the sugar content traits was relatively high (> 85%), except for sucrose (65%) (Table 3). The estimate of σ_G_^2^ was highly significant (P < 0.001) for all traits. Heritability of brix (> 95) was much higher than observed by Sandlin et al. [21] (~20). However, the latter study included two different locations, whereas the current study measured data at a single location over two years.

**Table 3:** Mean value, variance components and heritability for sugar content traits of the 97103 × PI 296341-FR watermelon RIL population in two years

**Traits**	**Env**	**Parental lines**		**RILs**			**Parameter**			
		**97103**	**PI296341**	**Mean**	**Min**	**Max**	**Genotype (G)**	**GEI**	***h***^***2***^**%**	**95% CI on *h***^***2***^
**Brix**	2010	11.0	0.8	3.45	0.50	8.80	3.42 ± 0.49^**^	0.12 ± 0.03^**^	96.94	95.47–97.93
	2012	10.2	1.3	3.46	0.58	8.60				
**Fructose**	2010	30.0	4.0	15.32	1.57	40.19	63.98 ± 10.00^**^	11.92 ± 2.17^**^	89.21	84.06–92.70
	2012	32.5	3.9	18.51	5.92	43.47				
**Glucose**	2010	17.5	2.0	9.65	1.05	28.08	24.11 ± 3.94^**^	5.87 ± 1.14^**^	85.7	78.87–90.32
	2012	20.0	1.5	12.42	2.85	29.62				
**Sucrose**	2010	45.2	0.4	1.97	0.08	20.50	4.79 ± 1.09^**^	3.35 ± 0.75^**^	64.59	47.68–76.03
	2012	40.2	0.1	0.94	0.10	15.56				

^*^, ^**^indicate significance at P < 0.05 and 0.01, respectively. GEI: Genotype by Environment Interaction; CI: confidence intervals. The unit of Brix is degress and unit of fructose, glucose and sucrose content is milligram per gram fresh fruit.

Significant (P < 0.001) genotype by environment interactions (GEI) for traits were also observed. GEI variance was primarily due to differences in the magnitudes of the genetic variances among the environments, rather than a lack of genetic correlations between environments. Significant positive correlations (P < 0.001) were observed for the phenotypic and genetic correlations among traits across two environments (Table 4).

**Table 4:** **Phenotypic (*upper value*) and genetic (*lower value*) correlations among sugar content traits for 97103 × PI 296341-FR watermelon RIL population across two years**

	**Brix**	**Fructose**	**Glucose**	**Sucrose**
**Brix**	0	0.92^**^	0.88^**^	0.65^**^
**Fructose**	0.95^**^	0	0.92^**^	0.55^**^
**Glucose**	0.93^**^	0.92^**^	0	0.42^**^
**Sucrose**	0.81^**^	0.67^**^	0.56^**^	0

^**^indicate significance at P < 0.01.

### QTL detection for sugar traits in the 97103 × PI 296341-FR population

Two, five, one and three QTL were detected for brix, fructose, glucose and sucrose, respectively based on single environment analysis (Table 5 and Figure 1).

**Table 5:** Main features of the QTL for traits based on single environment (Env) analysis

**QTL**	**Flanking marker**	**Position (cM)**	**CI**	**Env**	**Add effect**	**R**^**2 **^**(%)**
**Brix**						
*Qbrix2-1*^M^	2Bin3-2Bin4	15.9	13.9-18.3	2012	0.95	23.37
***Qbrix2-2***^**M**^	2Bin20-2Bin21	71.9	68.9-76.1	2010	1.16	29.3
***Qbrix2-2***	2Bin20-2Bin21	71.9	68.9-76.1	2012	0.92	31.37
**Fructose**						
*Qfru2-1*^M^	2Bin3-2Bin4	15.9	13.9-18.3	2010	4.38	23.46
*Qfru2-3*	2Bin10-2Bin11	36.9	35.9-39.9	2012	3.75	28.07
***Qfru2-2***^**M**^	2Bin20-2Bin21	74.9	68.9-76.1	2010	3.20	23.05
***Qfru2-2***	2Bin20-2Bin21	72.9	68.9-76.1	2012	3.08	28.16
*Qfru6*^*M*^	6Bin5-6Bin6	18.4	17.4-21.8	2010	2.60	21.97
*Qfru8*^M^	8Bin13-8Bin14	24.3	22.3-27.6	2012	3.96	15.25
**Glucose**						
*Qglu6*^M^	6Bin5-6Bin6	19.4	17.4-21.8	2010	2.70	22.55
**Sucrose**						
*Qsur2-1*^M^	2Bin3-2Bin4	15.9	13.9-18.3	2010	0.92	10.14
*Qsur2-2*^M^	2Bin20-2Bin21	71.9	68.9-76.1	2010	1.03	13.77
*Qsur5*^M^	5Bin5-5Bin6	19.1	15.1-19.3	2010	0.81	10.77

QTL in bold were detected in both years. CI: confidence intervals. Add effect: positive additive effect means parental cultivar 97103 contributed the favorable alleles.

#### QTL for brix

Four QTL were detected for brix using joint analysis (Figure 1; Table 6), with additive effects ranging from 0.41 to 0.83 and R^2^ values from 8.28 to 28.87%. Two of the QTL (*Qbrix2-1, Qbrix2-2*) are located on chromosome 2 and both have R^2^ > 20%. As expected, the elite parental cultivar (97103) contributed the favorable alleles at all loci.

**Table 6:** Main features of the QTL for traits based on joint analysis across two environments

**QTL**	**Flanking marker**	**Position (cM)**	**CI**	**Add effect**	**R**^**2 **^**(%)**
**Brix**					
*Qbrix2-1*	2Bin3-2Bin4	15.9	14.9-18.3	0.83	22.14
*Qbrix2-2*	2Bin20-2Bin21	73.9	69.9-76.1	0.54	28.87
*Qbrix6*	6Bin9-6Bin10	35.5	34.5-40.5	0.41	8.28
*Qbrix8*	8Bin13-8Bin14	24.3	22.3-27.6	0.79	13.71
**Fructose**					
*Qfru2-1*	2Bin3-2Bin4	17.9	13.9-18.3	3.30	25.95
*Qfru2-2*	2Bin20-2Bin21	75.9	71.9-76.1	2.11	24.55
*Qfru3*	3Bin24-3Bin25	64.7	61.7-65.2	1.12	5.86
*Qfru6*	6Bin6-6Bin7	23.8	21.8-27.1	2.33	15.93
*Qfru8*	8Bin13-8Bin14	25.3	22.3-27.3	3.52	14.9
**Glucose**					
*Qglu6*	6Bin6-6Bin7	23.8	21.8-27.1	2.51	16.71
**Sucrose**					
*Qsur2-1*	2Bin3-2Bin4	15.9	14.9-18.3	0.82	6.39
*Qsur2-2*	2Bin20-2Bin21	71.9	68.9-76.1	0.76	9.65
*Qsur5*	5Bin5-5Bin6	19.1	16.1-19.3	0.54	7.97

CI: confidence intervals. Add effect: positive additive effect means parental cultivar 97103 contributed the favorable alleles.

#### QTL for fructose

Five different QTL (Table 6 and Figure 1) associated with fructose were detected on chromosome 2 (*Qfru2-1, Qfru2-2* and *Qfru2-3*), chromosome 6 (*Qfru6*), and chromosome 8 (*Qfru8*) using joint analysis. R^2^ values ranged from 5.86% to 25.95%. Two of the fructose QTL on chromosome 2 (*Qfru2-1* and *Qfru2-2*) co-localized with QTL associated with brix (*Qbrix2-1* and *Qbrix2-2*).

#### QTL for glucose

Only one QTL was detected for glucose according to single environment analysis, as well as on the joint analysis across environments (Table 5, Table 6 and Figure 1). This QTL (R^2^ = 16.71) co-localized with the QTL for fructose on chromosome 6.

#### QTL for sucrose

Three different QTL associated with sucrose were detected on chromosome 2 (*Qsur2-1* and *Qsur2-2*) and chromosome 5 (*Qsur5*) based on single and joint analysis. *Qsur2-1* and *Qsur2-2* were located in the same chromosomal regions where the brix (*Qbrix2-1* and *Qbrix2-2*) and fructose (*Qfru2-1* and *Qfru2-2*) QTL mapped (2Bin3-2Bin4 and 2Bin20-2Bin21).

The allele from the parental cultivar 97103 was associated with higher sugar content for all the loci detected.

### Integration of QTL information

The 58 QTL previously reported for 12 traits [21-23] (Additional file 1: Table S5) and the 13 newly identified QTL for brix, sucrose, fructose and glucose content (Table 6, Additional file 1: Table S5) were placed onto the integrated map (Figure 1). QTL described in Sandlin et al. [21] were re-named to reflect chromosomal location in the integrated map (and thus genome sequence) rather than the linkage groups of the original study (Additional file 1: Table S5, Figure 1). The integration of all the QTL on the integrated map makes it possible to more precisely compare the location of QTL mapped in different populations and to use the physical map locations of the markers to identify candidate genes for specific traits.

Comparison of brix QTL in accession-specific linkage maps showed brix QTL located on chromosomes 1, 2, 6, 7 and 8 (Additional file 1: Table S5, Figure 1). Three different QTL on chromosome 2 were associated with brix in the three different genetic backgrounds (elite × citron, elite × egusi and elite × elite). *Qbrix2-2* identified in 97103 × PI 296341-FR and QBRX2-2 identified previously in the KBS × NHM were the only QTL for brix that were co-localized across populations. The possible explanation is that the variability of brix in the different genetic backgrounds are largely controlled by different loci, including major QTL with large effects on chromosome 2 and a number of QTL with lower effects on chromosomes 1, 6, 7 and 8.

One of the major brix QTL (*Qbrix2-2*) was co-localized with major QTL for fructose (*Qfru2-2)* and sucrose (*Qsur2-2*) in 2Bin20-2Bin21. The 2Bin20-2Bin21 region is in a large interval of about 4 Mb containing 416 genes. The other major brix QTL (*Qbrix2-1*) that co-localizes with fructose (*Qfru2-1*) and sucrose (*Qsur2-1*) QTL in the 2Bin3-2Bin4 region spans an interval of 2.3 Mb and contains 234 genes. The mean glucose content (0.94 - 1.97, Table 3) in the RIL population was much lower than the fructose (15.32 - 18.51, Table 3) and sucrose content (9.65 - 12.42, Table 3). The possible reason why brix QTL was co-localized with fructose and sucrose QTL is that fructose and sucrose are the main components of total sugar content (brix), while the glucose content can only contribute a little to total sugar accumulation.

The major QTL for seed size (*Q100swt6*, *Qsl6* and *Qswd6*, Additional file 1: Table S6) identified by Prothro et al. [22] in the elite × elite (flanking markers NW0251236 and NW0250242) and elite × citron (flanking markers NW0248118 and NW0248583) populations co-localized on the integrated map (Additional file 1: Table S1 and S5). The regions between the flanking markers spans 1.37 Mbp and 0.64 Mbp in the two populations respective, with a 0.62 Mbp overlap, containing 58 predicted genes.

In addition to comparing the location of QTL across different populations, the integrated map is also useful to identify potential additional polymorphic markers in important chromosomal regions. The egusi (*eg*) locus associated with the high oil, edible egusi-type seed was previously mapped between marker NW024835 and NW025024 [23]. This 20.9 cM section represents a 4.28 Mbp segment in the draft genome, containing 246 genes. The integrated map now gives us the opportunity to use the additional 11 SSR and 2 InDel markers in this region (Additional file 1: Table S1) to narrow the region of interest by potentially mapping these markers in the population of interest.

Interestingly, 12 economically important QTL corresponding to 6 fruit traits [brix, fruit length (FL), fruit width (FWD), fruit weight (FWT), rind thickness (RTH) and fruit shape index (FSI)] were detected on chromosome 2 (Additional file 1: Table S5, Figure 1). One possible explanation for the co-localization of fruit size and fruit sugar content QTL is that fruit maturity was a confounding factor, especially in populations that segregate widely for time to fruit maturity. However, both the current study and Sandlin et al. [21] tried to limit this potentially confounding factor. It remains to be seen whether fruit maturity played a role in co-localization for fruit sugar content and fruit size QTL.

## Conclusions

In this study, we developed an integrated map which consisted of 698 SSR, 219 InDels, 36 SV and 386 SNP markers from four independent mapping populations, which includes the three subspecies of watermelon. The integrated map described herein enhances the utility of genomic tools over previous watermelon genetic maps, such as those used in map-based cloning and sequenced scaffolds anchoring and orientation. A large proportion of the markers in the integrated map are SSRs, InDels and SNPs, which are easily transferable across laboratories. Moreover, the populations used to construct the integrated map include genotypes in broad horticultural groups (elite, citron and egusi type), guaranteeing the future utility of the markers in a broad range of cultivars and experimental crosses. The high marker density of the map allows for the selection of specific markers to customize mapping and molecular breeding applications, that will be useful for positional cloning of important genes, identification of QTL, MAS, the development of novel genetic stocks (e.g., nearly isogenic lines and inbred backcross lines), analysis of germplasm and detection of commercial hybrid seeds [10].

Fifty-eight previously reported quantitative trait loci (QTL) for 12 traits were integrated into the map and 10 new QTL for sugar content were identified. The positioning of economically important QTL in the integrated map facilitate comparative QTL analyses among populations of different origins to provide deeper insights into the genetic control of the diverse phenotypic variability observable in watermelon germplasm. For example, QTL for brix on chromosome 2 co-localize with QTL associated with FWT, FL, FWD, RTH and FSI, suggesting, perhaps, the existence of pleiotropic effects with fruit maturity. Multi-population analysis is a more powerful approach for detecting QTL/candidate gene associations. For instance, in the two major QTL on chromosome 2 involved in brix (sugar accumulation), annotated genes involved in polysaccharide metabolism, transport and corresponding regulation genes may become candidate genes for those QTL [26].

## Methods

### Mapping populations

Four previously described mapping populations derived from independent crosses were used to develop the integrated map (Table 1). An F_8_ population consisting of 103 recombinant inbred lines (RILs) derived from a cross between the elite Chinese line 97103 and the U.S. Plant Introduction (PI) 296341-FR [20], and an F_2_ population consisting of 182 individuals derived from a cross between the elite ZWRM50 and PI 244019 (ZWRM × citron) [21] were both elite × citron populations. In addition, an elite × elite [Klondike Black Seeded × New Hampshire Midget (KBS × NHM)] RIL population consisting of 164 lines, and an elite × egusi [Strain II × PI 560023 (SII × egusi)] F_2_ population consisting of 187 individuals were also included [21]. Total DNA was isolated from expanding leaves of three-week old plants using the modified CTAB method [27].

### Construction of the integrated map

The molecular markers used for integrated map construction included 698 SSR, 219 InDel, 36 structure variation and 388 SNP markers [20,21]. Because the majority of the markers were mapped in the 97103 × PI 296341-FR RILs population, we chose this population as reference for integrated map construction. In order to integrate all the markers accurately, we obtained all the published 388 SNP marker genotypes in the 97103 × PI 296341-FR RILs population from GBS (genotyping by sequencing) as described by Guo et al. [28] (the other GBS data of this population has not been published). The previous map [20] was designated the “skeleton bin map” and was used in further marker integrations. We then constructed the integrated map by adding SNP markers to the skeleton bin map. SNP markers were added to corresponding bins in JoinMap 4.0 [29] with minimum likelihood odd (LOD) score equal to 4. A bin signature comprises the integrated segregation pattern of marker loci, which do not recombine in the RIL population and are thus incorporated in the bin as described in Ren et al. [30].

Marker segregation distortion in each of the mapping populations was investigated employing Joinmap 4.0 software [29]. The segregation ratios of markers in the population were examined by Chi-square analysis in Joinmap 4.0. Markers with segregation ratios that differed from expected 1:1 at P < 0.001 were classified as segregation distortion markers. Regions larger than 10 cM in the original map or spanning ten loci showing significantly skewed segregation were defined as segregation distortion regions (SDRs). Colinearity was compared using colinearity figures drawn with CIRCOS software (http://circos.ca/software/).

### Sugar content phenotypic data

The 97103 × PI 296341-FR RIL population and parents were evaluated in Beijing (39.48°N, 116.28°E) over two years (2010 and 2012) in a randomized complete block design with two replications. Each year was considered as an environment. In order to ensure the fruits were ripe, we divided the RIL population into early-maturing, mid-maturing and late-maturing subgroups according to previous maturity data of each RIL line. The early-maturing and mid-maturing fruits were harvested 30 and 35 days after manual pollination, respectively. The late-maturing citron-type fruits were harvested 40–45 days after pollination to ensure that they were mature. Each fruit was cut lengthwise and only the centre of the fruit was used for sugar content measurements. Degrees brix (BRX) was measured using a pocket refractometer pal-1 (ATAGO Co., Ltd., Tokyo, Japan) from a sample of juice collected from the center of each watermelon. Subsequently, the center juice of the same watermelon was used for detecting fructose, sucrose and glucose content by High Performance Liquid Chromatography (HPLC) (LC-10A VP SHIMADIU Co., Ltd., Japan) [31].

### Statistical analysis of phenotypic data

The analysis of variance (ANOVA) was conducted using PROC GLM in SAS8.0 [32]. The broad-sense heritability (*h*^*2*^) for each trait was calculated on a per plot basis as *h*^*2*^ = *σ*_*G*_^2^/(*σ*_*G*_^2^ + *σ*_*GE*_^2^/*n* + *σ*_*e*_^2^/*nr*), where σ_G_^2^, σ_GE_^2^ and σ_e_^2^ were the variance estimates of genotypic, genotype by environment interaction and experimental error; n and r were the number of environments and number of replications. The *h*^*2*^ confidence intervals (CI) were calculated according to Knapp et al. [33]. The Pearson’s phenotypic correlation coefficients among traits across all environments were calculated on a mean basis using the SAS PROC CORR [32]. The genetic correlations among traits were conducted with PLABSTAT software (https://plant-breeding.uni-hohenheim.de/software.html).

### Sugar content QTL detection and integration of previous QTL

In our study, two mapping analyses were carried out as follows: (1) analysis for each single environment (each year); (2) joint analysis across all environments. The single environment and multi-environment joint analyses were performed using the QTL Network ver. 2.0 software [34] based on a mixed-model based composite interval mapping (MCIM). Mixed-model based composite interval mapping was carried out by using forward–backward stepwise regression with a threshold of P = 0.05 to select cofactors, and the window size set at 10 cM. The threshold for declaring the presence of a significant QTL was defined by 1000 permutations at a significance level of P = 0.05. The confidence interval calculated by the odds ratio reduced by a factor of 10 was averaged for each of the QTL according to Yang et al. [35]. The final genetic model incorporated significant additive and epistatic effects, as well as their interactions with environments. QTL detected in different environments for the same trait were considered to be the same if their confidence intervals overlapped.

Previous QTL were integrated into the map within the marker intervals according to the location presented in the original publications [21-23]. For illustration purposes, graphic representation of the two flanking markers’ LOD support interval was defined as the associated QTL’s position. In order to provide visual images of marker’s genomic positions, integrated markers and QTL were plotted using Mapchart 2.2 (http://www.wageningenur.nl/en/show/Mapchart.htm) [36].

## Authors’ contributions

YR participated in integrating of map and QTL data, QTL mapping of the sugar content trait, writing the draft manuscript. CM provided the three (KBS × NHM, SII × Egusi and ZWRM50 × PI 244019) population’s SNP marker information and QTL data, critical revision and wrote part of this manuscript. GG planted the 97103 × PI 296341-FR RIL population and managed this procedure in the greenhouse. YZ performed statistical analysis of phenotypic data. HZ constructed the 97103 × PI 296341-FR RIL population. SG and HS carried out the BLAST analysis of SNP marker’s sequences. JZ collected the phenotype of sugar content in 97103 × PI 296341-FR population. YX participated in the design and management of the study, wrote and revised the manuscript. All authors read and approved the final manuscript.

## Supplementary Material

Additional file 1: Table S1 SSR, InDel, SNP and SV markers used to construct the integrated genetic map of watermelon. Their chromosome, bin position, genetic distance (cM), marker name, repeat motif, scaffold name, primer start, primer end, production size from 97103, the SNP marker sequence and primer sequences are listed. **Table S2.** The elite × elite [Klondike Black Seeded × New Hampshire Midget (KBS × NHM)] RIL population based SNP genetic map. **Table S3.** The genetic map of elite × egusi [Strain II × PI 560023 (SII × Egusi)] 187 individuals F_2_ population. **Table S4.** The elite × citron (ZWRM50 × PI 244019) F_2_ population genetic map. **Table S5.** The mapped QTL and corresponding intervals in the integrated map. Click here for file

Additional file 2: Figure S1 (A) Colinearity of marker’s order in individual watermelon genetic maps and integrated genetic map in chromosomes 2 to 11. (B) Colinearity of locus order among three genetic maps, integrated watermelon genetic map (GM) and physical map (PM) in chromosomes 2 to 11. Population codes K_LG, S_LG, Z_LG corresponding to KBS × NHM, StrainII × PI 560023 (egusi), ZWRM50 × PI 244019 (citron) genetic maps [21]. Click here for file
