# Supplementary figures and images for: An integrated genetic map based on four mapping populations and quantitative trait loci associated with economically important traits in watermelon (Citrullus lanatus)
Source: BMC Plant Biol. 2014 Jan 20;14:33. doi: 10.1186/1471-2229-14-33 (PMC3898567; doi:10.1186/1471-2229-14-33)

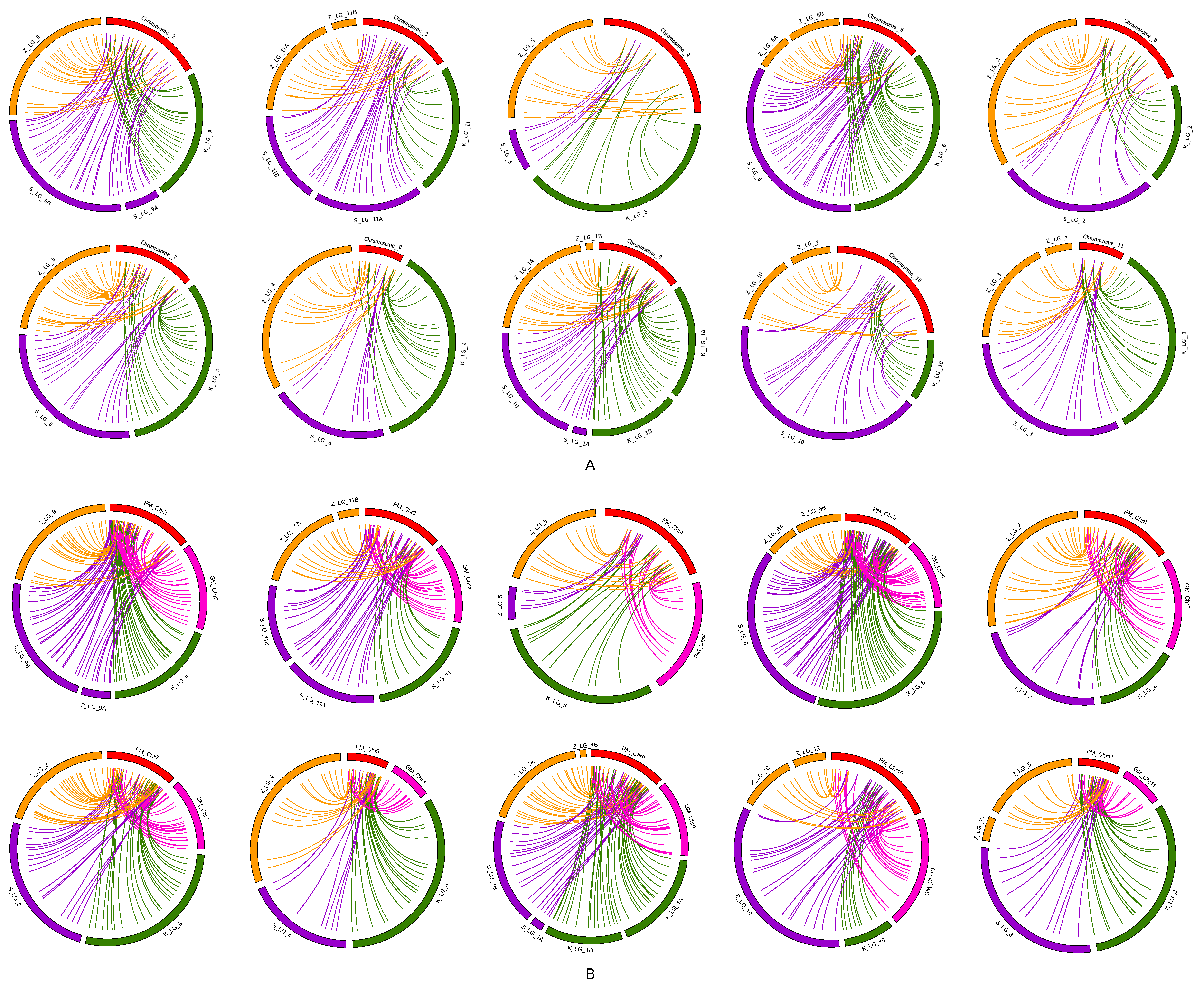

Supplement: Additional file 2: Figure S1 — (A) Colinearity of marker’s order in individual watermelon genetic maps and integrated genetic map in chromosomes 2 to 11. (B) Colinearity of locus order among three genetic maps, integrated watermelon genetic map (GM) and physical map (PM) in chromosomes 2 to 11. Population codes K_LG, S_LG, Z_LG corresponding to KBS × NHM, StrainII × PI 560023 (egusi), ZWRM50 × PI 244019 (citron) genetic maps [21]. [file 1471-2229-14-33-S2.tiff]
